# Modeling Human Sexual Motivation in Rodents: Some Caveats

**DOI:** 10.3389/fnbeh.2019.00187

**Published:** 2019-08-27

**Authors:** Olivia Le Moëne, Anders Ågmo

**Affiliations:** Department of Psychology, University of Tromsø, Tromsø, Norway

**Keywords:** sexual motivation, sexual behavior, orgasm, ejaculation, paracopulatory behavior, lordosis

## Abstract

Sexual behavior is activated by motivation. An overwhelming majority of experimental studies of the intricacies of sexual motivation has been performed in rodents, most of them in rats. Sometimes it is desirable to generalize results obtained in this species to other species, particularly the human. It is hoped that studies of the neurobiology of rodent sexual behavior may shed light on the central nervous mechanisms operating in the human, and the search for efficient pharmacological treatments of human sexual dysfunctions relies partly on studies performed in rodents. Then the issue of generalizability of the rodent data to the human becomes crucial. We emphasize the importance of distinguishing between copulatory acts, behavior involving the genitals, and the preceding event, the establishment of physical contact with a potential mate. Comparisons between the structure of copulatory behavior in rats and humans show abysmal differences, but there may be some similarity in the underlying mechanisms. The endocrine control of sex behavior is shortly mentioned, and we also compare the effects of the few drugs known to affect both rodent and human copulatory behavior. The stimuli activating sexual motivation, often called desire in the human literature, are examined, and the sexual approach behaviors in rats and humans are compared. There is a striking similarity between these species in how these behaviors respond to drugs. It is then shown that the intensity of sexual approach is unrelated to the intensity of copulatory behavior. Even though the approach is a requisite for copulation, an activity that requires at least two individuals in close physical contact, these two aspects of sexuality do not covary. This is similar to the role of the testosterone in men and male rats: although the hormone is needed for sex behavior, there is no correlation between serum testosterone concentration and the intensity of copulation. It is also pointed out that human sexual behavior is mostly determined by social conventions, whereas this is not the case in rats and other rodents. It is concluded that some observations in rats can be generalized to the human, but extreme caution must be exercised.

## Introduction

The typical textbook definition states that motivation is a concept referring to the mechanisms responsible for the activation, direction and persistence of behavior. According to this definition, the organism would be completely inactive in the absence of motivation. Once the organism has been activated, motivational systems determine which of all possible behaviors should be performed, and for how long the organism should persist with that behavior. Thus, motivation is underlying all activity and the choice of the specific activities to be performed at any moment. It is difficult to imagine a more fundamental concept in the science of behavior. These basic notions have been extensively discussed elsewhere (Hernández-González et al., 2008; Ågmo, 2011).

The early search for understanding motivational processes concentrated on rather basic behaviors, such as drinking, eating, and sex. It was believed that the motivational control of these basic behaviors was similar in all animal species. Consequently, the choice of species as an experimental subject was often based on convenience. However, already in 1949, at a meeting with the American Psychological Association, Frank Beach expressed concern about the overly frequent use of rats, hamsters and guinea-pigs in behavioral research (Beach, 1950). He feared that the concentration on a few, similar species, was incompatible with a real comparative psychology, and would make it impossible to determine if and how behavioral principles established in one species were at work in other species. The question of the generalizability of observations in one species to another is still unresolved.

In the present review article, we will discuss the generalizability of observations made on rat sexual behavior to the human. In other words, we will ask the question of whether we can use rat sex as a model of human sex. Some general notions about rat models and their potential utility have been outlined elsewhere (Ågmo et al., 2004; Ågmo, 2014), and they will not be repeated here. Instead, we will provide an in-depth analysis of the usefulness of observations of copulatory behavior on one hand and of sexual approach behaviors on the other, in rats and humans. Long ago, it was pointed out that the validity of generalization between species is strictly dependent on the quality of the description and understanding of the behavior in each of the species we want to generalize between (Beach, 1976). Therefore, we will include an analysis of the characteristics of rat and human sexual behavior. We will also discuss similarities and differences in the endocrine control of sexual behavior, and of the effects of drugs on these behaviors. In the end, we will ascertain that we are not now in possession of sufficient data of sufficient quality for any firm conclusion. Before turning to these issues, however, we will define the essential concepts employed here. This should reduce the possibility of misunderstanding and enhance clarity of all subsequent arguments.

## Definitions

Sexual motivation, often called sexual desire in the human literature, is an abstract concept referring to the *probability of displaying copulatory behavior when a mate is available or the intensity of that behavior when displayed*. It can also refer to the *intensity of approach to a potential sexual partner*. Since sexual activities (except masturbation) require at least two individuals in close physical proximity, any sexual encounter is preceded by approach behaviors.

The intensity of copulatory behavior can be quantified in many ways in male rats. We consider short latencies to mount, intromission or ejaculation as well as large number of mounts and intromissions as indicators of high intensity, whereas long latencies and low numbers indicate low intensity. High copulatory rate (number of sexual acts per unit time) and short interintromission intervals can likewise be considered indicators of high intensity, and low rate and long intervals constitute evidence for low intensity. In female rats, the indicators range from lordosis quotient and number of paracopulatory behaviors in the standard observation cage and these plus the temporal aspects of interaction with the male in the divided cage and seminatural environment (see “Rodents” section for explication of the terms used). In humans, the intensity of copulatory behavior is rarely defined or quantified. It appears that simple self-report of the number of copulatory encounters per unit time is used as indicator of the intensity of that behavior. Throughout this article, we refer to one or several of the abovementioned criteria whenever we mention the intensity of copulation. The intensity of sexual approach behaviors in rats and humans will be operationally defined in the “Sexual Approach Behaviors” section.

Copulatory behavior *is any action leading to sexual reward*. Sexual reward is a state of positive affect activated by physical stimulation of the genitalia or mental representations of such stimulation (Ågmo, 2007, p. 3). Evidence for the capacity of mental representations to cause sexual reward indistinguishable from that obtained by genital stimulation is limited to the human female. Fantasies alone can lead to the subjective experience of orgasm and the physiological manifestations of that state identical to those observed after orgasm caused by clitoral stimulation (Whipple et al., 1992). Likewise, imaging studies have revealed that the brain areas activated by fantasies or clitoral stimulation are similar (Wise et al., 2016). In men as well as in males and females of non-human species, physical stimulation of the genitals seems to be required for the obtainment of sexual reward.

It may appear inadequate to consider fantasizing leading to orgasm as a copulatory behavior. However, the fantasies are often about genital interaction with a mate (Seehuus et al., 2019), i.e., about copulatory behavior in a strict sense. The fact that the mate is imaginary rather than real is not crucial, according to our judgment. It may also be argued that humans may engage in sexual activities without obtaining or expecting to obtain sexual reward. In those cases a different reward, for example money, improved relationship or favors of all kinds, operates. Thus, motor patterns similar to those constituting copulatory behavior become instrumental for obtaining non-sexual reward. We do not consider such behavior as sexual. It may also be noted that these behaviors probably are determined by motives other than sexual. In fact, Meston and Buss (2007) have listed more than 200 possible motives, most of them unrelated to sexuality, for engaging in motor patterns similar to copulation.

We prefer the term “copulatory behavior” rather than “sexual behavior”, since the former more explicitly refers to genital activities. Another reason for avoiding “sexual behavior” is to clearly distinguish between non-genital sexual approach behaviors and acts involving the genitals. Thus, from here on we stick to “copulatory” instead of “sexual” when referring to behaviors involving the genitals.

**Figure 1:**
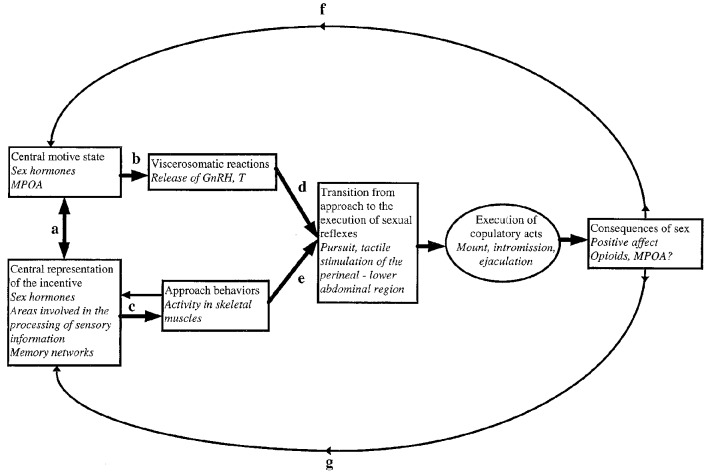
A model for sexual incentive motivation. The text in italics represents the example of the male rat. **(a)** A reciprocal excitatory relationship functioning in such a way that the central motive state enhances the sensory system’s sensibility to stimuli with sexual significance. When such stimuli are perceived, the sensory system excites the central motive state which in turn further sensibilizes the sensory system, i.e., the relationship is one of reciprocal positive feedback. **(b)** At a certain threshold level of activity, the central motive state engages a series of viscerosomatic activities preparing the subject for sexual interaction. **(c)** The appropriate environmental stimuli activate motor patterns that bring the subject in contact with the source of stimulation. During approach, additional incentive stimuli may be encountered. These will be centrally represented and enhance the central motive state through **(a)**. **(d,e)** Provided that approach behaviors have been successful and that appropriate viscerosomatic reactions are being accomplished, the subject’s behavior may change from unconditioned or conditioned instrumental responses to the execution of sexual reflexes. These are activated by tactile stimulation of the perineal or lower abdominal region. If the subject is sexually inexperienced such stimulation is obtained accidentally. If the subject already has acquired sexual experience, then conditioned instrumental responses may facilitate the attainment of tactile stimulation necessary for activation of sexual reflexes. At the point of transition from approach to execution of copulatory reflexes, the behavioral sequence is aborted in the absence of tactile stimulation. In case that sexual reflexes indeed are activated, sex behavior will normally continue until ejaculation. **(f)** The positive affect induced by ejaculation will feed back to the central motive state where a short-lasting inhibitory system is activated. **(g)** At the same time, the positive affect and associated processes of reinforcement will strengthen the learning of associations between itself and environmental cues. These cues will acquire incentive properties in relation to the intensity of the positive affect that is experienced. For further details, see Ågmo (1999, 2011) and Paredes and Ågmo (2004). Reprinted from Ågmo (1999). Copyright (1999), with permission from Elsevier.

A model of the relationship between external stimuli, central nervous processes and somatic as well as visceral responses to these stimuli is presented in Figure 1. Much of the ensuing discussion is based on this model.

## Animal Sex Is Not Always a Model

For a long time, copulatory behavior in rats, hamsters, guinea-pigs, rabbits and many more exotic species was studied without any explicit intention of generalizing to other species. The basic purpose of these studies was simply to describe the nature of copulatory behavior and the internal and external stimuli controlling it in a particular species and even in particular strains of some species. Species and strain comparisons were frequent, but generalizations from one species or strain to another were made only with great caution (for example see Beach, 1976) or not at all. The influential normative descriptions of male rat sexual behavior (e.g., Beach and Jordan, 1956; Larsson, 1956) were never intended to be generalized to other species. The detailed analysis of the circuits and hormones controlling female rat lordosis is perhaps a still better example of this. The sensory pathways transmitting the stimulus required for activating the behavior, from the cutaneous receptors to the diencephalon, as well as the descending output to *musculus longissimus lateralis* and *musculus transversospinalis*, both responsible for the lordosis posture, have been painstakingly described (Pfaff, 1980). This is also the case for the action of the ovarian hormones in hypothalamic structures, down to the molecular level (reviewed in Pfaff, 2017; see also Micevych and Sinchak, 2018). The applicability of these findings to humans was not of any major concern to the brilliant scientists behind these discoveries. The rat was not used as a model for something; it was studied in its own right. Whether the knowledge about the molecular actions of steroid hormones are applicable to other animals, including humans, is a completely different and perhaps irrelevant question in this context.

## Why Do We Need Models for Studying Human Sexual Behavior and Motivation?

Satisfaction of scientific curiosity, for example understanding the intricacies of rat copulatory behavior and its hormonal control, is not of basic importance for organizations financing research or for scientists with utilitarian inclinations. To both of them, the use of non-human animals is a means of enhancing human well-being. Then, the discoveries made in non-human animals are of interest only if applicable to humans. Moreover, the problems addressed should preferably be related to important public health issues. Since sexual dysfunction neither is a cause of death nor of great expenses to society, research on such dysfunctions is not necessarily of high priority. Nevertheless, sexual activities have been reported to positively contribute to human well-being, as assessed by different types of questionnaires (Blanchflower and Oswald, 2004; Cheng and Smyth, 2015; Kashdan et al., 2018). Disorders of sexual function can lead to reduced quality of life (Hisasue et al., 2005; Rosen and Bachmann, 2008; Rosen et al., 2009). Thus, even though these disorders are not life-threatening, they may disrupt the life of the affected individuals.

The most common of the sexual disorders in women is sexual interest/arousal disorder (West et al., 2008; Burri and Spector, 2011). Before the DSM-5 (American Psychiatric Association, 2013) this condition was known as female hypoactive sexual desire disorder. We will use this old name. In men, the prevalence of hypoactive sexual desire disorder is somewhat below that of erectile dysfunctions and premature ejaculation (Beutel et al., 2006; McCabe and Connaughton, 2014). The opposite condition, hyperactive sexual desire, was rejected for inclusion in the DSM-5, but is nevertheless of some clinical concern (Kafka, 2014). It is often assumed that the paraphilias are associated with hyperactive desire, and treatments reducing desire may be viable therapeutic approaches to this kind of disorder (Kafka, 2003). The high prevalence of the reduced desire disorders and the social apprehension caused by the paraphilias, notably pedophilia, have prompted a search for efficient pharmacological treatment. This search was also inspired by the commercial success of treatments for erectile dysfunction. Regardless of the reasons behind the pursuit of drugs able to stimulate low sexual desire and to inhibit excessive desire, the need for preclinical models with acceptable predictive validity became apparent.

Other human sexual dysfunctions that have been modeled in non-human animals include premature ejaculation, a condition common in young men. Even though the role of sexual motivation in the etiology of premature ejaculation is unclear, this is another example of the search for a treatment of a sexual disorder using rodent models.

An entirely different condition, persistent lack of sexual attraction or asexuality, has attracted some attention during the last few decades. It has been estimated that between 0.4% and 3.3% of the adult population consider themselves as asexual (Aicken et al., 2013; Höglund et al., 2014). The condition is not included in diagnostic manuals like the ICD-11 or DSM-5 and is often regarded as a sexual orientation or identity (e.g., Hinderliter, 2013; Bogaert, 2015). There are, nevertheless, reports showing that some male rats and mice also may display a persistent lack of sexual attraction (Portillo and Paredes, 2003; Portillo et al., 2013). However, asexuality is not a clinical condition and consequently there is no interest in developing treatments. This means that there is no need for rodent models. The condition will not be further discussed.

## Human Copulatory Behavior

### Generalities

Even though Moll (1897) and Ellis (1933) had analyzed human copulatory behavior in elegant ways, the groundbreaking work of Kinsey et al. (1948, 1953) can be considered the origin of modern enquiries into human sexuality. Since the times of Kinsey, scientists have reported quantitative data concerning most aspects of human sexual behavior. The overwhelming majority of these data stems from self-reports of sexual activities. The Kinsey group obtained their data through highly structured interviews performed by well-trained interviewers whereas most of the subsequent work has been based on the use of questionnaires. Answers have been provided in either written form (e.g., Alexander and Sherwin, 1993; Merghati Khoei et al., 2018) or as responses to questions made over the telephone (e.g., Lewin et al., 1998). More recently, internet-based questionnaires have become widespread (e.g., Ritter et al., 2018). Regardless of the way in which the self-reports are obtained, they are notoriously unreliable. The most eloquent example of this is that men systematically report a considerably higher number of heterosexual partners than women. However, when a man has sex with a new woman, there is always a woman having sex with a new man. Thus, in societies where the proportion of men in the population is approximately equal to that of women, which is the case in most societies, the number of partners must be close to equal for the two sexes. This has been pointed out many times (e.g., Smith, 1992; Wiederman, 1997). Possible causes for the discrepancy between men and women in reporting the number of partners may be different accounting strategies (women counting, men estimating) and misreporting due to social norms, among others (Mitchell et al., 2019). In any case, the questionnaire-based notion that men are more promiscuous than women survives facts showing that it is impossible.

Since most of the knowledge about human copulatory behavior is based on questionnaires, it must be considered as approximate, in the best of cases. There are, however, notable exceptions. Masters and Johnson (1966) made careful observations of humans during actual copulation, and their work is still unsurpassed. Others have studied genital arousal (erection and vaginal lubrication) under various conditions, thereby obtaining objective data on sexual responses. Still, others have analyzed cerebral blood flow or oxygenation when humans are exposed to sexually relevant stimuli (e.g., Mouras et al., 2003), or during masturbation (e.g., Stoléru et al., 2012) while in a magnetic resonance scanner. Due to the constraints of the scanner tube, brain imagery during actual copulation has not been performed. Nevertheless, the imaging studies have given rise to sophisticated models of the cerebral control of human sexual behavior (e.g., Georgiadis and Kringelbach, 2012). However, the fact that a brain area is activated or inhibited during sexual activities does not constitute evidence for that area actually being important for these activities. Lesions in some of the areas showing intense fos activation during female rat sexual behavior can leave the behavior unaffected (Guarraci et al., 2004). This can be an example of the typical redundancy of brain systems mediating basic behaviors. The functions of one area can be fulfilled by other areas when needed.

Despite the fact that a large quantity of descriptive and a limited amount of experimental data concerning human copulatory behavior are available, we are seriously lacking knowledge about many basic aspects of that behavior. This becomes particularly evident as soon as we are interested in the mechanisms activating the behavior. Neither the central nervous mechanisms underlying human sexual motivation nor the stimuli that render a human attractive to other humans are more than vaguely understood. Since sexual motivation is activated by stimuli emitted from other individuals, knowledge of these stimuli and how they affect the receiving individual are essential. It must be observed that even if humans sometimes replace the external stimuli from another individual with mental representations of such stimuli, the mechanisms activating sexual motivation are probably similar.

### Description

**Figure 2:**
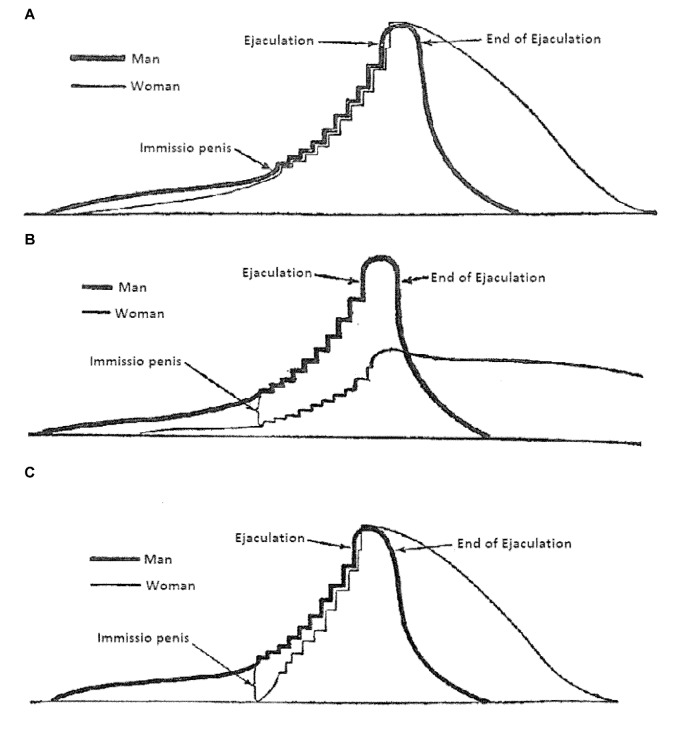
van de Velde’s (1926) illustration of human coital interactions. **(A)** The changes in sexual excitation during an ideal copulatory encounter. With ideal it is understood that the man and the woman reache orgasm at the same time. Excitation is defined as the summation of sexual desire and pleasure, bodily and psychic. **(B)** Similar to panel **(A)**, but here the sexual interaction occurs with an inexperienced woman without adequate coital stimulation. **(C)** Similar to panel **(A)**, but now the woman is sexually experienced. The prelude was omitted, but the woman’s low initial excitation was compensated for by her experience. From van de Velde (1926).

In his classical description of human copulatory behavior (van de Velde, 1926), it was assumed that this behavior was a continuous activity, starting with sexual arousal (erection and vaginal lubrication) followed by vaginal penetration and male thrusting until ejaculation in the male and orgasm in the female. van de Velde’s (1926) graphical illustration of human sexual intercourse is shown in Figure 2. The continuous nature of human copulation was later confirmed by direct observations (Masters and Johnson, 1966). In fact, these scientists adapted van de Velde’s (1926) scheme of copulation in their famous three-phase model (excitement, plateau, orgasm). A more recent account, based on clinical experience, added desire as an event preceding excitement (Kaplan, 1979), but the notion of a continuous process has not been challenged. The continuous flow of sexual behavior patterns in human encounters have been brilliantly described (Schick et al., 2016), although the descriptions are based on self-reports rather than on direct observation.

Classical accounts of human sexual activities only considered heterosexual encounters in pairs. Sex among groups of humans as well as copulation in same-sex pairs has not been studied and analyzed with the same care. As far as we know, however, the continuous nature of the interaction is still present, probably even in groups where the members may change partner in the midst of copulation (Tewksbury, 2002; Friedman et al., 2008; Meunier, 2014). Nevertheless, it can be maintained that the vast majority of human sexual activities occurs in heterosexual pairs, and that vaginal—penile intercourse is the most common of these activities (Laumann et al., 1994; Lewin et al., 1998). In fact, 95% of adult men and women reported to have engaged in penile–vaginal intercourse during the last 3 months regardless of whether the survey was made in Germany or Australia (Rissel et al., 2014; Goethe et al., 2018). None of the studies mentioned inquired about continuous or interrupted sexual encounters, probably because it is *a priori* assumed that human sexual encounters indeed are continuous.

### Endocrine Control

In men, there is clear-cut evidence for the crucial importance of androgens, acting on the androgen receptor, for the activation and maintenance of adequate sexual functioning (Bagatell et al., 1994; Schmidt et al., 2009). Estrogens are not required (Sartorius et al., 2014). In women, it is not clear if estrogens coming from the ovaries and from aromatization of androgens in other tissues, acting on estrogen receptors, or androgens, mainly coming from the adrenal cortex, and acting on androgen receptors, are needed. There are strong proponents for both opinions (Waxenberg et al., 1959; Tuiten et al., 2000; Cappelletti and Wallen, 2016). However, recent data showing the efficiency of testosterone therapy for treating low sexual motivation in women may settle the issue in favor of actions at the androgen receptor (Traish et al., 2009; Guay and Traish, 2010; Khera, 2015).

### Drugs and Sex

There is no lack of anecdotal evidence for the most spectacular drug effects in humans, but there are very few controlled studies. Still worse, the results of these studies are often contradictory. In fact, there are very few drugs for which there is solid evidence for some effect on human copulatory behavior. We will now examine these drugs. We do not consider the drugs improving erection as drugs modifying copulatory behavior, even though they make that behavior possible.

The time from vaginal penetration until ejaculation is called the intravaginal ejaculation latency. Some men ejaculate with a very short latency. Even though this is an expression of normal interindividual variation, it is considered a pathology with the label premature (early) ejaculation. A drug, dapoxetine, prolongs this latency in men diagnosed with premature ejaculation (Yue et al., 2015; Russo et al., 2016). The drug is, in fact, the only pharmacological treatment for premature ejaculation approved in Europe and in many other countries (excepting the United States).

Dapoxetine is a fast-acting specific serotonin reuptake inhibitor (SSRI). Not surprisingly, some of the SSRIs used for the treatment of depression have also been employed for the treatment of premature ejaculation, with results equally good or better than those reported for dapoxetine (reviewed in Waldinger, 2007). It is noteworthy that the ejaculation-delaying effect is desirable in men suffering from premature ejaculation, while it is regarded as an unpleasant side effect in men taking SSRIs for the treatment of depression. In fact, iatrogenic (caused by a presumably therapeutic treatment) sexual dysfunction is considered a serious problem with the SSRIs, and it is sometimes supposed to be the most frequent cause for abandoning treatment (e.g., Kennedy and Rizvi, 2009). This assertion, however, has no support in clinical data. Non-sexual side effects or lack of antidepressant effect are the main causes for discontinuation of treatment with the SSRIs (Bull et al., 2002). Nevertheless, deleterious effects of these drugs on sexual functions are not uncommon. All facets of sexuality, from desire through arousal to orgasm, have been reported to be affected by the SSRIs, in both men and women (reviewed in Rosen et al., 1999; La Torre et al., 2013). Delayed ejaculation in men and anorgasmia in women might be the most common adverse effects, but the poor quality of the clinical data precludes any firm conclusion (Kronstein et al., 2015). Indeed, in a carefully conducted, double-blind study on healthy, young men fluoxetine had no significant effect on any parameter of sexual function (Madeo et al., 2008). It appears that the effects of SSRIs on human sexual performance are inconsistent.

### Multiple Orgasms and Ejaculations

We have not been able to find any experimental data concerning the latency to orgasm from the moment of penile penetration into the vagina until orgasm in women. However, orgasm induced by masturbation (clitoral stimulation) has been carefully studied. The mean orgasm latency is usually around 7 min, and the duration of orgasm is about 20–30 s when objective measurements (change in vaginal blood flow or vaginal and anal contractions) are used (see Levin and Wagner, 1985, and references therein). Interestingly, self-reports of orgasm duration did not correlate with the physiological measurements, prompting Levin and Wagner (1985; p. 439) to remind us of the fact that “data obtained …… from questionnaires or interviews have suspect validity.”

There are many reports of women experiencing multiple orgasms in the course of a single sexual encounter (see Darling et al., 1991). Estimates of the proportion of multiorgasmic women range from 42.7% in the Darling et al.’s (1991) study to 14% in Kinsey et al.’s (1953) classical study. The interval between successive orgasms varies between a few seconds and a few minutes, and the number of sequential orgasms varies between 2 and 20 (Kinsey et al., 1953; Darling et al., 1991). The duration of a sexual encounter, from vaginal penetration until the last orgasm, is not known.

In healthy, young men the mean intravaginal ejaculation latency has been found to be 3.01 min (Kreutzer et al., 2001) in one study and 5.4 min in another (Waldinger et al., 2005). It appears that most sexual encounters end after the first ejaculation, although there are scant data supporting this assertion. In any case, detumescence follows ejaculation, and there is a period of time, called the post-ejaculatory refractory period, during which another erection is impossible.

The fact that there are almost no studies of the “refractory period” in men, has not impeded scientists from publishing reviews of the subject with irregular intervals (e.g., Levin, 2009; Seizert, 2018). One of the few published experimental studies used young men as subjects. They were asked to watch a pornographic video while erection (tumescence and rigidity) was monitored (Ekmekçioğlu et al., 2005). When erection was complete, the men applied mechanical stimulation to the penis until ejaculation. The mean ejaculation latency (time from the start of stimulation until ejaculation) was 2.2 min, not dramatically different from that measured in copula. The sexually relevant stimulation (pornographic video) continued after ejaculation. About 80% of the men showed complete detumescence after ejaculation, whereas the remaining proportion showed only partial detumescence. However, 68.2% of the men showed a second erection, indistinguishable from the first. The mean interval between ejaculation and the following erection was 19 min. Other studies employing a similar procedure have reported mean post-ejaculatory refractory periods of 11 (Aversa et al., 2000) and 13.8 (Mondaini et al., 2003) min. However, in these studies, the subsequent erection was not detected by objective procedures. The subjects themselves judged when it occurred.

Considering that a majority of men are able to have a new erection a couple of minutes after ejaculation, and that women may experience many orgasms in rapid succession, we need to explain why sexual encounters usually terminate after the man’s first ejaculation. Many explanations have been launched, but none is beyond the stage of speculation ( for a good example, see Turley and Rowland, 2013). It must also be mentioned that human copulatory behavior in informal settings, such as sex clubs, have been reported to consist of a series of ejaculations with different partners in men, and sequential orgasms with different partners in women. Also in the latter cases, the cause for ending copulatory activity remains unknown.

We propose that a very simple mechanism, negative alliesthesia, can offer a conceptual, but not neurobiological, explanation. Briefly, alliesthesia refers to the frequently observed fact that exposure to a reward momentarily reduces the value of that reward. For example, rats and humans like sweet solutions, and avidly consumes such solutions when made available. If they are pre-exposed to a small amount of the solution, they will consume far less than when non-exposed (Cabanac and Duclaux, 1973). Although negative alliesthesia first was reported for tastants, it also operates for other kinds of stimuli (Brondel and Cabanac, 2007). In the context of sex, having achieved one ejaculation or orgasm may reduce the reward value of sexual activity, and consequently the incentive value of sexually relevant stimuli. Thus, sexual activity ceases. Some humans may require more prolonged sexual activity before the negative alliesthesia has built up to the level required for ceasing sexual activity, and therefore continue copulating beyond the first ejaculation. The mechanisms underlying sexual alliesthesia are unknown, but the present notion provides at least a conceptual framework for the pursuit of these mechanisms.

Negative alliesthesia should not be confounded with habituation. The latter phenomenon requires repeated exposure to a constant stimulus, whereas negative alliesthesia may occur after a single exposure, as in humans ceasing to copulate after one orgasm. Furthermore, habituation is a case of non-associative learning, whereas alliesthesia refers to change in the reward value of a stimulus. However, in multiple ejaculators, like male rats, habituation to a female probably contributes to the end of sexual activity. In humans, this is probably not the case.

## Rodents

Copulatory behavior in rodents consists of a series of stereotyped motor patterns performed in an ordered sequence. Since the rat is the most studied and still most used species, we will limit the following description to male and female rats. Sexual behavior in mice, hamsters, and guinea pigs are somewhat different, but in all these species it is still a series of stereotyped motor patterns, and the central nervous control of this behavior is quite similar.

### Description: The Female Rat

The basic element of female rat sexual behavior is the lordosis posture, a concave arching of the back, stretched hind-legs, and the tail moved to one side (Pfaff et al., 1973). This posture exposes the vaginal opening, making it possible for the male to achieve vaginal penetration, in the rat literature called intromission. We will consistently use the term intromission when talking about copulatory behavior in rodents. In addition to lordosis, the female will often rapidly shake her head up and down and sideways, giving the impression that she wiggles the ears. Lordosis and ear-wiggling are activated by tactile stimulation from the male. Although stimulation of the flanks and rump is most efficient for activating lordosis, stimulation of any part of the body can be enough. There may also be some ear-wiggling without direct physical contact with the male. Finally, the female can approach the male, and then suddenly run away with darting or hopping movements. This behavior is called solicitation. The exact stimulus responsible for activating solicitation is unknown. Ear-wiggling and solicitation are frequently grouped together under the label paracopulatory or proceptive behavior (Erskine, 1989) Sexual encounters between a male and a female rat can be arranged in many ways. The most common is to put the animals together in a small cage and observe what they are doing. A variant is to divide the cage in halves with a wall having one or several holes. The size of the holes can be adjusted so that the slim female can move between halves whereas the fat male remains confined to one half. The female can thus escape from the male to her own half whenever she finds it convenient. An entirely different procedure is to create an environment somewhat similar to rats’ natural habitat. This can be done by combining a large open space with an artificial burrow, and allow a mixed sex group to live in the environment for some time. Such seminatural environments have been used only in a handful of studies of sexual behavior (reviewed in Chu and Ågmo, 2016b).

In the small cage, the members of the pair have no escape from each other. In the divided cage, the female has the privilege to escape from the male. It is often maintained that the female controls sexual interaction in this situation. In seminatural environments, both the female and the male can escape whenever they want, simply because of the size of the environment and the availability of easily defended nest boxes. In the latter situation, both males and females can and do control sexual interactions. If we want to study the ordered sequence of events constituting copulatory behavior, and obtain meaningful results, the small cage must be avoided. Since it fails to give the rats an opportunity to escape, and since escape is a fundamental part of sexual interactions among wild rats observed in their natural habitat (Calhoun, 1962; Robitaille and Bouvet, 1976), the small cage lacks external validity in the brunswikian sense. According to Brunswik (1955), an externally valid design should either be a random sample of experimental procedures in which the target event may occur or the test procedure should be as similar as possible to the subjects’ natural habitat (see also Petrinovich, 1980). Studies failing to incorporate at least one of these criteria lack external validity, and results cannot, therefore, be generalized beyond the specific procedure used. The divided cage and the seminatural environment offers surprisingly similar descriptions of the structure of female sexual behavior, and can probably be considered as externally valid.

**Figure 3:**
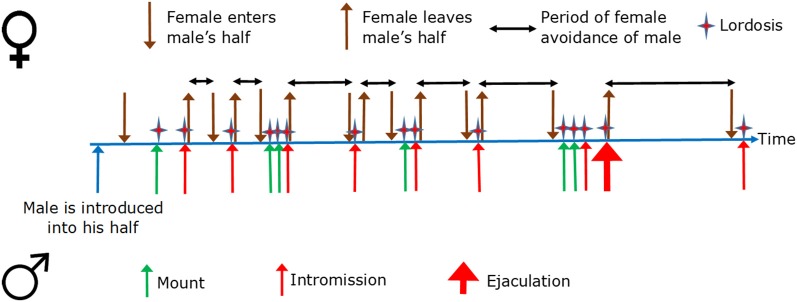
Sexual behavior in a cage in which the female can escape from the male copulation partner. The female is present already at the beginning of the timeline. The horizontal, double-ended black arrows represent the time the female has escaped from the male into her own half. The illustration ends after the first intromission following the first ejaculation. Copulation may have continued for several ejaculations, however.

In the divided cage, the female will sooner or later enter the male’s half, and the male will sooner or later mount the female. The mount may or may not be transformed into an intromission. If it is, the female will usually return to her half of the cage. If the mount ends without intromission, the likelihood for the female to escape to her own half is not above random (Ellingsen and Ågmo, 2004). In case the male ejaculates, the likelihood for the female escaping to her half of the cage is higher than it is after an intromission. Furthermore, the time she will remain in her half of the cage is longer than after an intromission. Thus, the likelihood of escape from the male and the time the female remains inaccessible are directly proportional to the intensity of sexual stimulation received (Erskine, 1989). Figure 3 illustrates the typical temporal sequence of female rat sexual behavior. The important thing to observe here is that female rat sexual behavior is a series of approach–avoidances.

Approach is activated by attractive stimuli whereas avoidance is a response to aversive stimuli. Therefore, during sexual interaction, the male is transformed from an attractive to an aversive stimulus by intromission and ejaculation. At the same time, intromission and ejaculation cause positive affect (see “Multiple Ejaculations and Orgasm” section). The mechanisms behind the contradictory reactions of the female are not entirely known, but some informed speculations have been made (Komisaruk and Whipple, 2000). One possible explanation is that mechanical stimulation of the vaginal wall momentarily reduces sexual motivation and causes short-lived pain.

In females in the seminatural environment, the interval to the next sexual event is less than 3 min after having received a mount. After an intromission it is about 5 min, and after an ejaculation it is about 13 min (Chu and Ågmo, 2014). During these intervals, the females are engaged in non-sexual activities or resting. These data show that sexual interactions in a seminatural environment have consequences similar to what was described for the divided observation cage. Despite the fact that three rather than one male were able to copulate with the female in the seminatural environment, the relationship between the amount of sexual stimulation received by the female and the interval to the next sexual event remained similar to that observed in the divided cage. Thus, female sexual behavior is a sequence of approach–avoidance also in a seminatural environment (Chu and Ågmo, 2014).

The female rat copulatory behavior pattern, lordosis, has a duration of 1–2 s (e.g., Ellingsen and Ågmo, 2004). In a seminatural environment, intact females display a total of about 200 lordosis during the period of behavioral estrus. This period has a mean duration of 7.3 h (Chu and Ågmo, 2014). For about 400 s of this time, the female is engaged in actual copulatory behavior, i.e., 0.015% of the time. The overwhelming majority of time was spent in other activities, unrelated to sex. These other activities were now and then interrupted by sexual acts. Data from a seminatural environment confirm that copulation in the female rat consists of a series of intermittent, short interactions with males.

### Description: The Male Rat

Turning to the male, we find the same sequence of approach–avoidance as in the female. In fact, it will soon become evident that there is a surprising similitude between male and female rat sexual behavior. Whereas the basic female sexual behavior pattern is the lordosis, the mount is the basic male behavior pattern. When mounting, the male stands on his hind legs with his forepaws placed on another rat’s rump from behind while performing a series of antero-posterior pelvic movements, thrusting. Accelerometric studies of the movements during copulation have shown that the mount is extremely stereotyped (reviewed in Moralí and Beyer, 1992) with a mean duration of about 400 ms and a thrusting frequency of about 18 Hz. During some mounts, the erect penis will make contact with the vaginal orifice. The male will then make a strong forward thrust leading to intromission. The duration of the intromission is about 400 ms. The male will thereafter dismount with a vigorous backward thrust. After a couple of intromissions, ejaculation will occur. Penile insertion lasts longer (1–2 s) and is accompanied by intravaginal thrusting and the expulsion of semen. The male dismounts slowly, without any backward thrust.

A mount not ending in intromission may be succeeded by another mount within a few seconds. An intromission will be followed by a short period of inactivity or non-sexual activities. In our laboratory, sexual quiescence after a mount bout with or without intromission lasts 42 ± 13.6 s (median ± semi-interquartile range), based on data from 143 rats tested in heterosexual pairs in a small cage. In these same males, quiescence following ejaculation lasted 301 ± 40.3 s. The conclusion to be drawn from this is that the period of sexual inactivity following a sexual interaction depends on the intensity of that interaction in males as well as in females.

**Figure 4:**
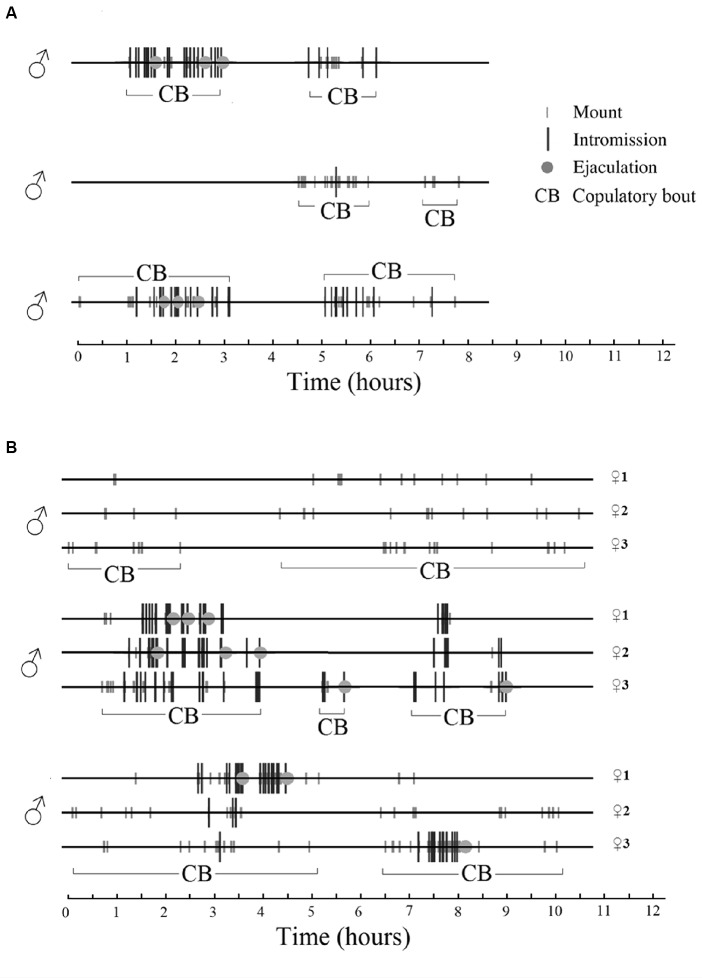
Sexual behavior displayed by male rats in a seminatural environment during females’ natural estrus. There were three males in the environment. Time 0 represents the beginning of estrus, i.e., when the female or females presented their first lordosis. A copulatory bout (CB) is a period of continuous (adjacent copulatory events are separated by less than 60 min) male sexual activity. **(A)** One single female is in estrus. All males copulate with the female during overlapping periods. **(B)** Three females are in estrus simultaneously. Each male copulates with the three females, and each female copulates with all the males in overlapping periods. Rat copulatory behavior seems to be entirely promiscuous, perhaps similar to what is observed in sex clubs frequented by humans (see “Multiple Orgasms and Ejaculations” section). For further details, see Chu and Ågmo (2015b). Reprinted with permission from the American Psychological Association.

In a seminatural environment, male sexual behavior is also a sequence of discrete events followed by long periods of non-sexual activities or complete inactivity. In fact, during periods of sexual activity the males spend 77% ± 4% of the time resting and grooming, while 8% ± 2% was spent on pursuing the female. Only 0.3% of the time was used for the execution of copulatory acts, i.e., mount, intromission and ejaculation (Chu and Ågmo, 2015b). Periods of sexual activity were defined as the time between the first mount or intromission recorded until the last copulatory event before a period of inactivity exceeding 60 min. An example of male sexual behavior in a seminatural environment can be found in Figure 4.

### Endocrine Control

It is established beyond doubt that gonadal hormones are required for the display of copulatory behaviors in male and female rats (for recent reviews, see González-Flores et al., 2017; Hull and Rodríguez-Manzo, 2017). In many strains of rats, simultaneous activation of both androgen and estrogen receptors are needed for male sexual behavior to occur. In female rats, androgen receptors do not contribute to sexual behaviors.

### Drugs and Rat Sex

Pharmacological studies of rat sex behavior were once upon the time very popular. We will make no intent to review the voluminous literature. This declining field has been reviewed many times before (e.g., Bitran and Hull, 1987; Paredes and Ågmo, 2004; Snoeren, 2015; Uphouse, 2014). Instead, we will focus on the few kinds of drugs having known effects on human copulatory behavior. These are, as mentioned, limited to dapoxetine and other SSRIs. Although the effect of flibanserin is questionable in humans, we will also mention the few studies performed in rats.

**Figure 5:**
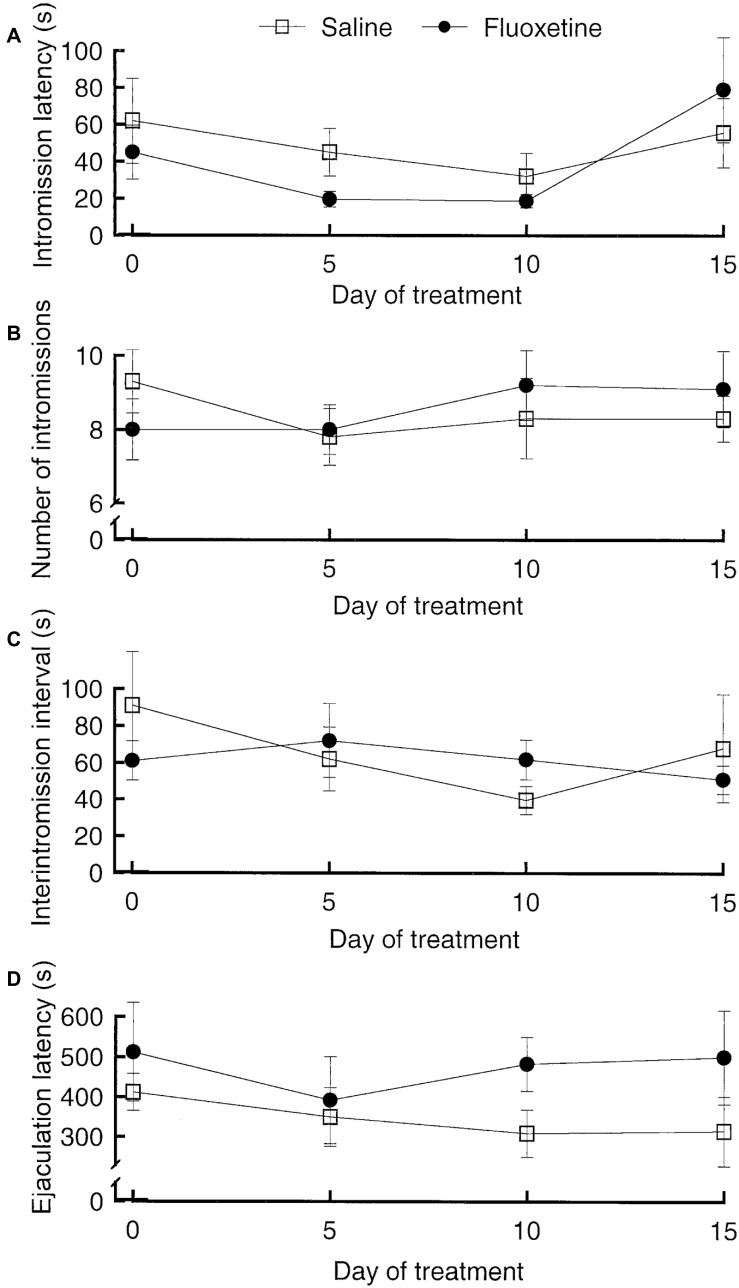
Parameters of copulatory behavior in male rats treated with fluoxetine, 10 mg/kg per day orally, or saline for 15 days. The test on Day 0 was performed 1 h after the first fluoxetine administration. **(A)** Intromission latency. Analysis of variance (ANOVA) for repeated measures on the factor Day of treatment and independent measures on the factor Treatment failed to reveal any statistically significant effect of Treatment, of Day of treatment and of the interaction Day × treatment (all *p*s > 0.07). **(B)** Number of intromissions. Also here, ANOVA failed to detect any significant effect (all *p*s > 0.51). **(C)** Interintromission interval. No effect (all *p*s > 0.39). **(D)** Ejaculation latency. No effect (all* p*s > 0.13). Data are mean ± standard error of the mean (SEM). Data are from an unpublished experiment, performed by one of the present authors (AÅ) together with Juoni Sirviö, Gro Sandberg and Live Sørensen.

The SSRIs fluoxetine and paroxetine have been shown to enhance ejaculation latencies in rats (e.g., Vega Matuszczyk et al., 1998; Waldinger et al., 2002). However, we were not able to see any effect of fluoxetine (Figure 5). This negative finding is in agreement with other studies (e.g., Frank et al., 2000). The only possible conclusion is that the effects of SSRIs in male rats are inconsistent. This is not surprising since only about 20%–30% of men treated with SSRIs for depression report sexual side effects and only part of those report delayed ejaculation. It is unlikely that prolonged ejaculation latency in such a small proportion of the experimental subjects could render the effect statistically significant.

Dapoxetine enhances the ejaculation latency in male rats as it does in men, but only in rats selected because of their initially short latency (Clement et al., 2012; Olayo-Lortia et al., 2015). For obvious reasons, there has been no interest in studying the effects of dapoxetine on female rat copulatory behavior.

There are several reports of reduced lordosis and paracopulatory behavior in female rats treated with fluoxetine (e.g., Matuszczyk et al., 1998; Maswood et al., 2008; Ventura-Aquino and Fernández-Guasti, 2013b). However, another SSRI, paroxetine, does not alter female rat copulatory behavior in any way (Kaspersen and Ågmo, 2012), not even after a very long treatment period (Snoeren et al., 2011). As was the case for the male rat, SSRIs have inconsistent effects on female copulatory behavior.

Flibanserin, an agonist at 5-HT_1A_ receptors and a weak antagonist at 5-HT_2A_ receptors, has been tested in rats. The drug enhanced the number of solicitations displayed by ovariectomized females after 2 weeks of treatment and on. Females treated with estradiol benzoate (EB) only or with EB combined with progesterone (P) responded in the same way (Gelez et al., 2013).

### Multiple Ejaculations and Orgasm

It is not known whether rats experience something similar to the human orgasm. Some argue that they do (Pfaus et al., 2016), whereas others consider it unnecessary to employ anthropomorphisms to explain rat behavior (e.g., Ågmo, 2007). Nevertheless, there are much data showing that sexual activity leads to positive affect in both male and female rats. Events or activities causing positive affect are considered to be rewarding. Sexual reward has been extensively studied with the conditioned place preference procedure, a procedure often used to evaluate positive affect induced by natural rewards as well as by drugs like morphine, cocaine and amphetamine. Ejaculation produces a robust place preference (e.g., Ågmo and Berenfeld, 1990). Several intromissions without ejaculation are also able to produce place preference, although ejaculation seems to be more efficient (Camacho et al., 2009; Tenk et al., 2009). Mounts without intromission are not enough. In the female, the receipt of several intromissions causes place preference, regardless of whether she copulates in the divided observation cage (Paredes and Alonso, 1997; Paredes and Vazquez, 1999) or in a small cage (Meerts and Clark, 2007, 2009b). Even artificial stimulation of the cervix works well in this procedure (Meerts and Clark, 2009a). It may be interesting to note that even prolonged copulation, leading to the receipt of several ejaculations, is just as rewarding as copulation limited to one ejaculation or 15 intromissions (Arzate et al., 2011). Regardless of whether the sexual reward experienced by rats have anything in common with the human experience of orgasm or not, we can conclude that sexual activities are rewarding for both rats and humans.

**Figure 6:**
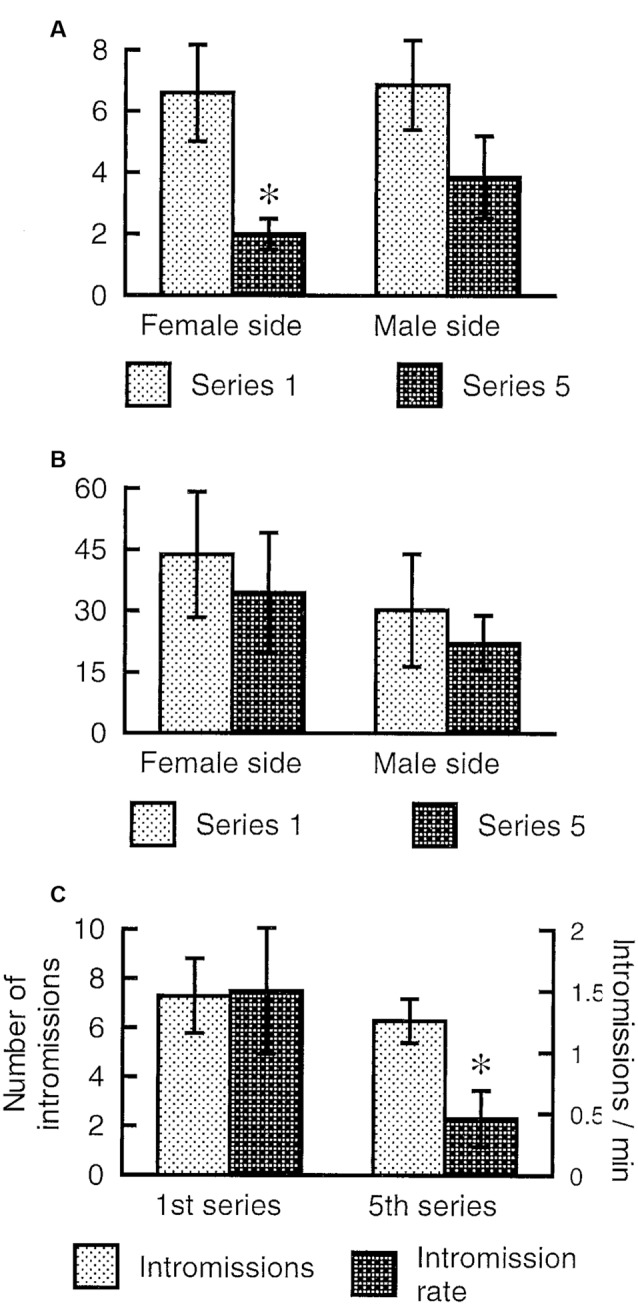
Paracopulatory behaviors displayed by female rats tested in a divided cage until the male had achieved five ejaculations. We determined the number of those behaviors displayed in the male’s half as well as the number displayed in the female’s half during the 1st and 5th ejaculatory series. **(A)** The rate expressed as number per minute of paracopulatory behavior. Two-factor ANOVA with repeated measures on one factor (Series) revealed a main effect of Series (*F*_(1,6)_ = 20.269, *p* = 0.004) but not of Side (female, male; *F*_(1,6)_ = 0.778, *p* = 0.412). The interaction Series × Side was also non-significant (*F*_(1,6)_ = 1.326, *p* = 0.293). **(B)** Number of behaviors. ANOVAs found no effect (all *p*s > 0.24). **(C)** Male copulatory behavior. The number of intromissions performed during the 1st and 5th series was similar (*t*_(6)_ = 0.786, *p* = 0.462, paired *t*-test). The rate of intromission, calculated as number per minute, was lower in the 5th series than in the 1st (*t*_(6)_ = 2.691, *p* = 0.018). Data (mean ± SEM) are from an experiment performed by Ellinor Ellingsen and Ana Lene Turi. *Different from 1st series (*p* < 0.05).

The female rat will display lordosis to every male mount during the entire period of behavioral estrus, and show undiminished amounts of paracopulatory behaviors until the abrupt end of estrus when observed in a seminatural environment (Chu and Ågmo, 2014, 2015a). In the divided cage, there is no reduction in lordosis responses after prolonged copulation, but the rate of paracopulatory behaviors is reduced when the female has received several ejaculations (Ventura-Aquino and Fernández-Guasti, 2013a). We have confirmed that observation in females having received five ejaculations during a prolonged test in a divided cage. However, although the rate of paracopulatory behaviors was lower in the 5th ejaculatory series than in the first, the number of these behaviors remained constant (Figure 6). Since the intensity of male behavior was much reduced in the 5th series, the interval between copulatory interactions increased, and the duration of the series was far longer than for the first series. Thus, even though a constant number of behaviors were displayed, the rate was inevitably reduced.

In conclusion, male and female rats continue to copulate for extended periods. The mechanisms underlying the end of a period of sexual activity remain obscure. In the male, the last event of a bout of copulatory activity is equally often a mount, an intromission or an ejaculation (Chu and Ågmo, 2015b). In the female, it seems that the end of sexual activity is associated with a sudden loss of attractivity to the males, at least in a seminatural environment (Chu and Ågmo, 2015a). As was the case with humans, the neurobiological mechanisms behind the end of copulatory activity in rats are unknown. Again, we propose that negative alliesthesia can be used as a conceptual basis for future work.

## Copulatory Behavior in Rats and Humans: Something in Common?

Concerning the structure of copulatory behavior, the differences between rats and humans are abysmal. Whereas a sexual encounter in rats consists of a series of very short periods of genital contact interrupted by long periods of non-sexual activities, in humans a sexual encounter is continuous, normally without any intrusion of non-sexual activities. The apparent dissimilarity in copulatory behavior may not apply to the underlying mechanisms controlling basic processes, though. In the human, ejaculation is triggered by continuous mechanical stimulation of the glans penis. In rats, the mechanical stimulation is intermittent. However, each intromission leads to a gradually increasing excitation, continuing to increase for several minutes post-intromission. The excitation is reinforced by the following intromissions until an ejaculatory threshold is reached (Larsson, 1960; extensively discussed in Ågmo, 2011). Thus, the difference between rats and humans is that in rats, a gradually increasing excitatory state is produced by intermittent mechanical stimulation whereas humans require continuous mechanical stimulation. In both species, ejaculation occurs when the excitation surpasses a threshold. Since the ejaculation latency is somewhat shorter in men than in rats, it appears that continuous stimulation causes a more rapid increase in excitation than intermittent stimulation does.

Male rats ejaculate many times before reaching sexual exhaustion, whereas most humans end a sexual encounter after the man’s first ejaculation. Whether this is a result of social learning or of the inherent nature of human copulatory behavior is not known, since the mechanisms causing cessation of copulatory activity are unknown. Likewise, it is not known if female rats experience something similar to orgasm in women. Consequently, we cannot know if rats, like women, may have multiple orgasms during a single sexual encounter.

Copulatory behavior is dependent on gonadal hormones in rats and humans, even though the crucial hormones may be different. In male rats, the simultaneous action of androgens and estrogens is necessary, whereas only androgens may be sufficient in men, as already mentioned. In female rats, estrogen and progesterone synergize to induce sexual behavior, whereas the role of these ovarian hormones is unclear in women, as pointed out above. Androgens do not contribute to female rat sexual behavior, but they may be important in women. There are also similarities in drug actions in rats and humans, despite the large differences in copulatory behavior. The most important similarities and differences in copulatory behavior between rats and humans are summarized in Table 1.

**Table 1:** Comparison of some basic characteristics of copulatory behavior in rats and humans and the responses to drugs used clinically for the treatment of sexual dysfunctions.

	Rats	Humans
General
Copulation is continuous	No	Yes
Copulatory motor patterns	Highly stereotyped	Extremely variable
Multiple ejaculations	The rule	Occasionally
Ejaculation latency^a^	~7 min	3–5 min
Post-ejaculatory period^b^	~5	~19 min
Multiple female orgasms	?	Yes
Latency to orgasm	?	~7 min^c^
Depends on gonadal	Yes	Yes
hormones^d^
Drug effects		
SSRI	May enhance ejaculation latency	Inhibition in some individuals
Dapoxetine	Enhances ejaculation latency	Enhances ejaculation latency
Flibanserin	Stimulates paracopulatory behaviors	Stimulates desire in women?

*?Unknown or uncertain effect; ^a^time from the first vaginal penetration until ejaculation in rats, time from penile insertion until ejaculation in men; ^b^time from ejaculation until the following vaginal penetration in rats, time from ejaculation until next erection in men; ^c^time from the start of clitoral stimulation until orgasm. Latency in copula is unknown. ^d^Males and females collapsed*.

## Sexual Approach Behaviors

We have already mentioned that copulatory behavior requires physical proximity of at least two individuals, and that copulation, therefore, must be preceded by approach behaviors. In fact, van de Velde (1926) described the first phase of a sexual encounter, the prelude, in the following words: “As soon as the first stirrings of the impulse of approach are perceptible, the prelude to sexual union begins” (from a reprint of the English translation, van de Velde, 1926, p. 102). This idea is not much different from Kaplan’s (1995) notion of the desire phase. For these and other reasons, we have suggested that the intensity of sexual approach behaviors is an exquisite indication of the intensity of sexual motivation (Ågmo et al., 2004; Spiteri and Ågmo, 2006; Ågmo, 2014). It has even been argued that the intensity of copulatory behavior is not an indicator of sexual motivation. In fact, Meyerson and Lindström (1973, p. 1) wrote: “However, the intensity of the copulatory act or the readiness to respond to mating attempts of another individual cannot be taken as a measure of sexual motivation. It is the eagerness to seek sexual contact, not the consummatory act which interests us.” This is certainly an exaggeration, but there is no doubt that studies of sexual approach are most informative in rodent studies (for a discussion, see Ågmo, 2014).

The approach behaviors are as variable in rats as they are in humans. A rat can walk, run, jump, swim or dig in order to approach a potential mate, and a human can engage in all kinds of activities with the purpose of establishing contact with and approach to a desired individual. This means that we cannot describe sexual approach behaviors in terms of particular motor patterns. Consequently, it seems reasonable to consider all actions leading to reduced distance to a potential mate as sexual approach behavior. It is, however, most important to distinguish sexual approach from non-sexual or social approach. van de Velde (1926) simplified the issue by making the rather grotesque assumption that any “stirring of the impulse of approach” is a manifestation of the desire to establish a sexual encounter. However, both humans and rats are social animals, and most approaches to other individuals are made because of purely social motivation. In rats, experimental setups can be arranged so that sexual approach can be clearly distinguished from social approach. To the contrary, in humans this distinction can rarely, if ever, be made. It could be assumed that a purely heterosexual woman approaches other women for uniquely social reasons. However, if she would approach a man, it could be either because of social motivation, sexual motivation, or a combination of both. In fact, the quantification of the intensity of human sexual approach behavior, uncontaminated by social approach, is extremely difficult or perhaps impossible. As we soon will see, this conundrum has been solved by replacing studies of human approach behaviors with studies of genital responses. Such responses cannot be regarded as manifestations of social motivation. To the contrary, it is generally accepted that they represent sexual motivation and nothing else.

### Sexual Approach in Men and Women

There are, for the reasons mentioned in the preceding section, no experimental studies of the behavior patterns employed by humans when sexually approaching other individuals. There are many literary or anecdotal descriptions, but the scientific value of these anecdotes is most doubtful. Likewise, the many manuals explaining how to successfully approach and seduce men or women are of little help for scientists. Nevertheless, there are some possibilities to objectively evaluate something that might approximate human sexual approach behavior.

As was outlined in the model of sexual motivation illustrated in Figure 1, a sexual incentive will activate approach behavior and visceral responses, provided the stimulus is presented in an adequate context. Among the most reliable visceral responses to sexual incentives is enhanced genital blood flow, manifested as erection in men and vaginal lubrication in women. Thus, the magnitude of the genital response can be used as a proxy for the impact of sexually relevant stimuli on sexual motivation. The latter is the factor causing the individual to engage in approach behaviors. It must be mentioned that in the human literature, the genital response to sexual incentives is called “sexual arousal.”

The complex relationship between the genital responses and the subjective experience of these responses, as reported on a questionnaire, is beyond the scope of the present discussion. It has been brilliantly reviewed elsewhere (Meston and Stanton, 2019). In our opinion, the notion of subjective sexual arousal does not contribute to our understanding of the mysteries of sexual motivation (Ågmo, 2008). It cannot be used for non-human animals, for example. In the following, we will use genital blood flow as the sole reliable indicator of sexual motivation or desire in the human.

### External Stimuli and the Activation of Sexual Motivation in Men and Women

We have already mentioned that humans may use mental representations (fantasies) of sexually relevant stimuli instead of external stimuli for the activation of sexual responses, including orgasm in women. There are also observations showing that fantasies make an important contribution to sexual desire (Birnbaum et al., 2019). Unfortunately, these private events are difficult to investigate with experimental methods, and are beyond the scope of the present communication. We will, therefore, only discuss external stimuli. The initial activation of sexual motivation, and consequently of sexual approach behaviors and genital responses, must be achieved by distant stimuli, i.e., olfactory, auditory, or visual. Once approach has been successful, tactile stimuli will become crucial for the further enhancement of sexual motivation and the eventual initiation of copulatory activities.

To our knowledge, there is only one study in which the stimuli important for sexual approach in the human has been described in a non-laboratory setting. The probability for a woman to be approached by a man in a nightclub depended on the amount of naked flesh exposed and the amount of sexually suggestive dance movements made (Hendrie et al., 2009). None of the other stimuli emitted by a woman, for example facial expressions or verbal activities, had any effect. This study seems to be unique in the way that direct approach rather than genital responses to sexually relevant stimuli was observed. However, the nightclub setting imposes many limitations, and experimental studies of actual approach behavior are desperately needed before any conclusion can be presented as to the exact stimuli causing this approach. In the meantime, we need to base our knowledge of the stimuli activating sexual motivation on studies of genital responses.

In both men and women, visual and auditory stimuli with sexual content are efficient for activating genital responses, and the combination of these modalities is still more efficient (McConaghy, 1974; Gaither and Plaud, 1997). In fact, moving pictures of diverse sexual activities, in heterosexual or homosexual pairs, and sometimes in groups, are routinely used in laboratory studies of genital responses. In vernacular language, this kind of movies are called pornographic. In the scientific literature, the euphemism erotic is often used, for some unknown reason. There is an extensive literature on the importance of the content in written descriptions of sexual activities or in pornographic movies, in relation to the sex of those depicted as well as of those observing, and of preferences for the own or the opposite sex (reviewed in Rupp and Wallen, 2008). We will ignore this literature, and simply conclude that the modalities of vision and audition are crucial in human sexual approach. There is no evidence for any role of olfactory stimuli, despite the widespread belief to the contrary (for discussion and references, see Ågmo, 2007; Le Moëne and Ågmo, 2017). We regret that this might be inconvenient for the perfume industry and for the sociobiologists.

It must be mentioned that neutral stimuli may acquire the capacity to activate genital responses through learning. In a most elegant study, the presentation of a neutral picture was associated with clitoral stimulation in women. Clitoral stimulation is an unconditioned stimulus causing enhanced vaginal blood flow. After a few pairings, the picture enhanced this blood flow by itself, i.e., it worked as a conditioned stimulus (Both et al., 2008, 2011). There are several other studies showing that classical conditioning can transform any stimulus into a sexually relevant stimulus in men and women (reviewed in Hoffmann, 2017). It is generally believed that this kind of learning is the basis of fetishism (Köksal et al., 2004). In any case, the fact that learning can make any stimulus capable of activating sexual motivation in humans should not be ignored.

### Drugs and the Activation of Sexual Motivation (Desire)

#### Women

The vaginal response to pornographic movies is not altered by menopause (Laan and van Lunsen, 1997; Suh et al., 2004), despite the strong reduction in circulating estrogens associated with that state. This observation can suggest that estrogens are not important for responses to sexually relevant stimuli, or that even in menopause they are above the level required for maximal responding.

One single drug (flibanserin, Addyi^®^) has been approved by the Federal Drug Administration for the treatment of female hypoactive sexual desire disorder. Initially, the drug was developed as an antidepressant, but it failed both some preclinical tests and a phase II study (Gellad et al., 2015). Since some of the participants in the clinical study reported heightened sexual desire, it was decided to develop the drug for treatment of low sexual desire rather than for depression. Although the phase III studies indeed suggested some effect on sexual desire (DeRogatis et al., 2012; Thorp et al., 2012), it is questionable whether this drug is superior to placebo (Saadat et al., 2017; Anderson and Moffatt, 2018). Perhaps this is related to the fact that flibanserin was approved because of political pressure rather than because of proven efficiency (Woloshin and Schwartz, 2016). Interestingly, the effects of flibanserin on vaginal responses to sexual stimuli has not been evaluated and, as mentioned, its effect on subjective measures, mainly self-reports, of sexual desire is questionable.

Another kind of drugs that might affect sexual motivation in women is the SSRI. As was the case with flibanserin, the effect of SSRIs on vaginal responses to sexually relevant stimuli has not been studied. Thus, we do not know if these drugs are affecting anything else than performance on questionnaires. It is amazing that the rather simple procedures needed for objectively assessing vaginal responses are so rarely used, whereas the notoriously unreliable questionnaires are omnipresent. Most unfortunately, we are constrained to conclude that the effects of clinically used drugs on vaginal responses to sexual stimuli are entirely unknown.

Even though adrenergic compounds are not used for the clinical treatment of sexual desire disorders, a nonspecific adrenergic α and β agonist, ephedrine, has been tested for effects on female sexual functions. The drug increases the vaginal response to pornographic movies (Meston and Heiman, 1998). To the contrary, an α_2_ antagonist, yohimbine, has no effect (Meston and Worcel, 2002). This is cumbersome, since blocking the α_2_ receptor generally enhances the release of noradrenaline (Gobert et al., 2004). Consequently, yohimbine and ephedrine should have similar effects. To further complicate things, it has been reported that the α_2_ agonist clonidine reduces the vaginal response to a pornographic movie (Meston et al., 1997). It seems that the role of the adrenergic receptors in sexual responses in women needs to be further evaluated before any conclusion can be reached.

#### Men

Seventy-five percentage of castrated men show a drastically diminished penile response to a pornographic movie segment (Greenstein et al., 1995). This is also the case in men suffering from severe hypogonadism after treatment with leuprolide (Schober et al., 2005), a compound inhibiting gonadotropin release from the pituitary. These observations suggest that androgens are needed for the activation of genital responses to sexually relevant stimuli, hence for the activation of sexual motivation.

Some SSRIs are used for treating paraphilia because it has been suggested that this condition may be related to unusually high levels of sexual motivation (e.g., Kafka, 2003). Although there are some data suggesting good effects (Briken and Kafka, 2007), there is no consensus regarding the long-term usefulness of SSRIs (Holoyda and Kellaher, 2016). This may be related to the observation that SSRIs like fluoxetine and citalopram do not alter the penile response to a pornographic movie in healthy young men, not even after 4 weeks of treatment (Haensel et al., 1998; Madeo et al., 2008). This interesting observation suggests that SSRIs do not reduce sexual desire, at difference to widely held beliefs. In fact, in the Madeo et al. (2008) study, the SSRIs not only failed to affect objectively measured sexual arousal but also sexual desire as evaluated by a questionnaire.

A review of sexual functions in people using drugs for recreational purposes, including tobacco and alcohol, concluded that not even these commonly used and socially acceptable drugs have been adequately studied and that no firm conclusion as to their sexual effects could be presented (Zaazaa et al., 2013). Therefore, we will end the account of drug effects here.

We cannot leave this section without addressing the limitations of using genital responses as a proxy for sexual approach behaviors. Even though we maintain that both genital blood flow and sexual approach are manifestations of sexual motivation, we must accept that these expressions of motivation do not always coincide. An eloquent example is the reliable, stimulating effect of sildenafil on penile responses to sexually relevant stimuli, such as pornographic movie segments, in men with (e.g., Gingell et al., 2004) and without erectile dysfunction (e.g., Kolla et al., 2010). However, there is no evidence showing that sildenafil enhances any other aspect of sexual function than erection (Jones et al., 2008). This means that we cannot automatically infer effects on sexual approach behaviors from effects on genital responses. Additional data are always required. Unfortunately, in the absence of experimental studies, these additional data have to come from self-reports or questionnaires of some kind.

### Sexual Approach in Rodents

**Figure 7:**
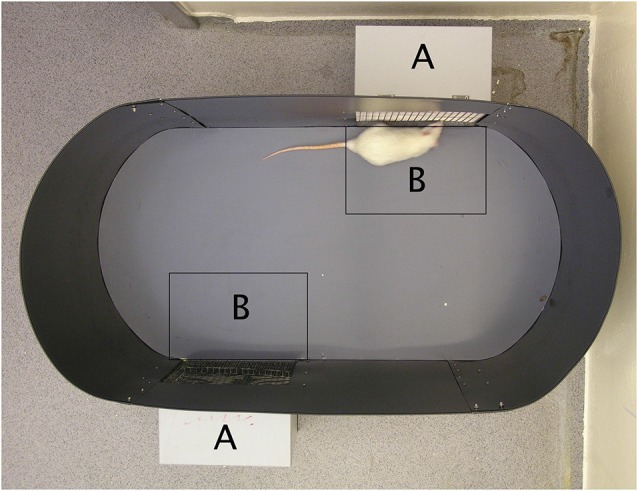
A photograph of the sexual incentive motivation test arena. The incentive animal cages (marked with an A on the photograph) are located on the outside of the oval arena (100 × 50 cm). They are detachable from the outside of the wall so that the position of the incentive animals can be changed randomly. The side facing the arena is made of a wire mesh that allows the experimental subject to see, smell and hear the incentives. A virtual zone of 21 × 29 cm (marked with a B on the photograph) is defined outside each incentive animal cage. A computerized videotrack system determines the experimental subject’s position, the time spent in the incentive zones, the number of visits to them, the distance moved during the test, the mean speed of movement while moving, and the immobility time. Reproduced from Spiteri and Ågmo (2006). Copyright 2006, republished with permission from Elsevier.

The direct observation of sexual approach behavior in rodents does not pose the slightest problem, and there are many established procedures available (reviewed in Ventura-Aquino and Paredes, 2017). We will briefly describe the one that we have used for many years (Ågmo, 2003; Ågmo et al., 2004). The setup is illustrated in Figure 7. It has been validated in several ways, and it allows for a clear distinction between approach behavior to a potential mate (sexual approach) on one hand and to a social stimulus (social approach) on the other. In order to determine whether the approach to the sexual incentive really represents sexual motivation, we performed a series of experiments. First, we replaced the sexual incentive with a second social incentive, so that the experimental male or female rat only had social incentives to approach. There was no systematic difference between these two incentives regardless of which specific incentive was used. In fact, all social incentives were about equally attractive. With the regular setup, with a sexual and a social incentive, we then tested male and female subjects that should have no sexual motivation. Castrated males did not distinguish between incentives, and when they were treated with testosterone, they enhanced the approach to the sexual incentive without altering approach to the social incentive (Ågmo, 2003; Attila et al., 2010). In females, there was no variation in approach to the social incentive during the estrus cycle, whereas approach to the sexual incentive peaked in proestrus. Ovariectomized females approached equally the sexual and the social incentive, whereas females given EB alone or EB + P approached the sexual incentive far more than the social incentive (Spiteri and Ågmo, 2006). Finally, we tested males that should have reduced sexual motivation because of immediately preceding sexual activity. Actually, the test was performed after that the males had had continuous access to a receptive female for 4 h. The males did not distinguish between the social and the sexual (a different female) incentive. Likewise, females tested immediately after having received three ejaculations did not approach the sexual incentive more than the social (Ågmo et al., 2004). All these observations made us conclude that the procedure indeed can be used for quantifying sexual motivation expressed as approach behavior.

#### The Stimulus Control of Rodent Sexual Approach

**Figure 8:**
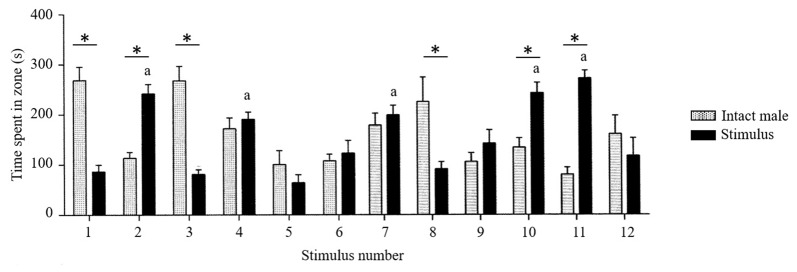
Sexual approach behavior in male rats in response to different kinds of stimuli (black bars). As control for social approach, an intact male was used (gray bars). The experimental subject could choose between spending its time close to the male stimulus or close to the alternative stimulus. The ordinate shows time spent in proximity to the different stimuli. The stimuli are indicated on the abscissa. 1. An intact male and an empty, clean cage, *N* = 11. The time spent close to the male was far superior to the time spent close to the empty cage. This illustrates nicely the male’s social attraction. 2. An intact male and a sexually receptive female, *N* = 10. The difference between the time spent with the female and the time spent with the male is the intensity of sexual attraction [(sexual + social approach) – social approach] = sexual approach. 3. Intact male and playback of female ultrasonic vocalizations, *N* = 10. As can be seen, the vocalizations were not more attractive than a silent, empty cage. 4. Intact male and the odor of a sexually receptive female, *N* = 11. The female had spent 6 h in the cage before being removed just before the test. She left behind urine, feces and other body odors that may stick to the floor and walls of the small cage. The odor was not more attractive than the male. Thus, odor by itself has no sexual attractant properties according to our definition (see Stimulus 2). 5. As always, an intact male. The other stimulus was here an anesthetized female and the experimental subject was anosmic. Thus, the only stimulus modality available to the male was vision, *N* = 9. Neither of the stimuli was more attractive than an empty cage. Thus, olfaction is necessary for social as well as for sexual approach. Visual stimuli have no impact. 6. Intact male and a devocalized female were the available stimuli, and the experimental subject was anosmic. The test was performed in complete darkness. Thus, neither visual nor olfactory stimuli were available, and no stimuli from the female’s vocal cords, *N* = 10. None of the stimuli was attractive to the experimental male. 7. Intact male and the odor of a female + playback of female ultrasonic vocalizations were the alternatives, *N* = 10. Both were equally attractive, showing that odor + vocalizations have no sexual attractant properties. 8. Intact male and anesthetized female + playback of vocalizations. The experimental subject was anosmic, *N* = 9. There was no difference between these latter stimuli and an empty cage, showing that the sight and vocalizations from a female does not attract a male at all. 9. Intact male and sexually receptive female. The experimental subject was anosmic, and the test was performed in complete darkness. The only stimulus available to the male was auditory, *N* = 10. They did not produce sexual attraction. 10. Intact male and anesthetized female, providing olfactory and visual stimulation but no sounds, *N* = 11. She was as attractive as an active female. 11. Intact male and devocalized female, test performed in complete darkness, *N* = 9. This female was as attractive as an intact female. 12. Intact male and devocalized female. The subject was anosmic, *N* = 10. No social or sexual attractivity was observed. *Different from the social incentive (male rat), *p* < 0.05. ^a^Different from the empty cage (stimulus 1), *p* < 0.05. The conclusion from this experiment was that olfactory stimuli need to be combined with some other stimulus in order to activate sexual attraction. The other stimulus cannot be produced by the female’s vocal cords. Further details can be found in Ågmo and Snoeren (2017).

Exactly as is the case in humans, sexual approach in rats must be activated by a distant stimulus. There is no reason to believe that rats produce mental representations of sexually relevant stimuli, meaning that any manifestation of sexual motivation must have its origin in an external stimulus. We have carefully determined the role of olfactory, auditory and visual stimuli (Ågmo and Snoeren, 2017) in the procedure described in the preceding section. The results of the corresponding experiment are shown in Figure 8. It turned out that olfactory stimuli are necessary but not sufficient. The odor of a sexual incentive is not superior to a social incentive. The odor employed was produced by a sexually receptive female left in the incentive cage for 6 h and withdrawn just before the test. Urine, feces and body odors left on the walls and floor were the odor sources. To become superior to a social incentive, odor must be combined with another stimulus, either auditory or visual. The exact auditory and visual stimuli required could not be identified, but we excluded the ultrasonic vocalizations that rats emit in the presence of conspecifics, particularly conspecifics of the opposite sex. Devocalized sexual incentives were not less approached than vocalizing incentives. The lack of importance of ultrasonic vocalizations had already been established in a series of studies in this same procedure (Snoeren and Ågmo, 2013, 2014a,b) and in a seminatural environment (Chu et al., 2017). It must be added that, as is the case in the human, any stimulus may acquire sexual significance through conditioning (see Kvitvik et al., 2010; Chu and Ågmo, 2012; and references therein). For a much more extensive analysis of the stimulus control of sexual approach behaviors, the reader is referred to Le Moëne and Ågmo (2017).

Even though not systematically evaluated in other rodent species, we assume that olfaction is of prime importance. The role of additional sensory modalities is not known.

#### Drugs and Sexual Approach in Female Rats

Because of scientists’ fascination for copulation, studies of sexual approach behavior in rodents are not often performed. For example, the only drug in clinical use for the explicit treatment of sexual motivation, flibanserin, has not been studied with regard to its effects on sexual approach. To the contrary, there are some data concerning the SSRIs. We have reported that 20 days of treatment with paroxetine reduces sexual approach in female rats (Kaspersen and Ågmo, 2012). Similar data have been reported after treatment with fluoxetine (Adams et al., 2012). This drug also affects female rat behavior in an operant procedure. The authors interpreted the effects as signs of reduced sexual motivation (Uphouse et al., 2015). Contradictory data have also been reported. In a study employing a procedure almost identical to ours, Matuszczyk et al. (1998) failed to detect any effect of fluoxetine on sexual approach, even though the drug reduced lordosis. Nevertheless, we conclude that the majority of data suggests that the SSRIs reduces sexual approach in the female rat.

#### Drugs and Sexual Approach in Male Rats

**Figure 9:**
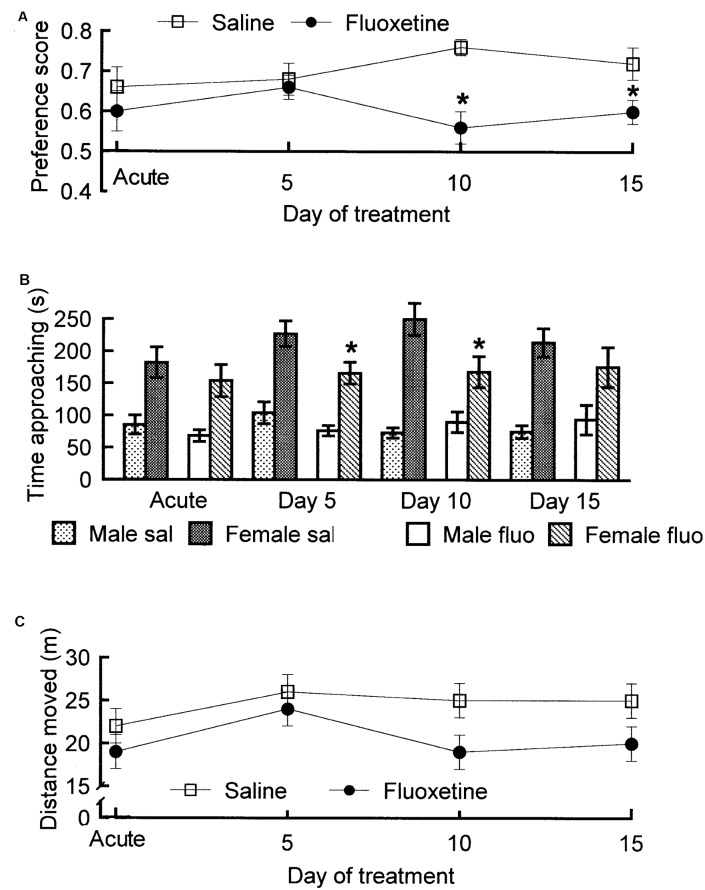
The effects of fluoxetine, 10 mg/kg and day given orally, on sexual approach behaviors in male rats. The procedure described in “Sexual Approach in Rodents” section was used. Test duration was 10 min. The acute test was performed 60 min after drug administration. **(A)** Preference score. Mixed two-factor ANOVA with Treatment and Day as factors revealed a significant Treatment effect (*F*_(1,34)_ = 7.528, *p* = 0.010), but there was no effect of Day (*F*_(3,102)_ = 0.192, *p* = 0.902) and no interaction Treatment × Day (*F*_(3,102)_ = 1.239, *p* = 0.300). **(B)** Time spent in the vicinity of the social incentive (another male) and in the vicinity of the sexual incentive (receptive female rat). Three-factor ANOVA with the factors Incentive, Treatment and Day found an effect of Incentive (*F*_(1,34)_ = 86.072, *p* < 0.001) and of Treatment (*F*_(1,34)_ = 7.134, *p* = 0.012) but not of Day (*F*_(3,102)_ = 1.789, *p* = 0.154). The time spent in the vicinity of the female was superior to that spent in the vicinity of the social incentive at all tests according to the Tukey HSD test. There was also an interaction between Incentive and Treatment (*F*_(1,34)_ = 7.993, *p* = 0.008). Fluoxetine did not alter the time spent in the vicinity of the male incentive whereas the time spent in the vicinity of the sexual incentive was reduced. There were no other interactions (*p*s > 0.300). Thus, fluoxetine did not modify social approach but reduced sexual approach. Since there was no interaction Treatment × Day, this effect was present already at the acute test. **(C)** The distance moved during the test, a measure of ambulatory activity, was not altered by treatment and did not vary between days (*p*s > 0.102). Thus, the inhibition of sexual approach cannot be explained as a secondary effect of reduced activity. Data are mean ± SEM. *Different from saline, *p* < 0.05. From an unpublished experiment performed by AÅ, Juoni Sirviö, Gro Sandberg and Live Sørensen.

As was the case with females, sexual approach behaviors have rarely been evaluated in drug studies in males. This makes it easy to summarize the literature, particularly since we will limit ourselves to clinically used drugs with established or presumed effects on sexual motivation in men. The only candidate drugs for inclusion in this group are the SSRIs, as mentioned. It has been reported that treatment of male rats with fluoxetine for 14 days reduces their approach to a sexually receptive female (Vega Matuszczyk et al., 1998). We have replicated this finding. As can be seen in Figure 9, fluoxetine treatment reduced sexual approach, particularly during the tests performed after 10 and 15 days of treatment. It seems that fluoxetine consistently reduces sexual approach in males.

### Sexual Approach in Rats and Humans: Any Similarities?

Table 2 summarizes the main characteristics of sexual approach behaviors in rats and humans. As can be seen, there are striking similarities. The main difference is with regard to the stimulus modalities involved in the activation of sexual motivation, hence approach. Furthermore, it is likely that mental representations of sexually relevant stimuli as sources of sexual motivation are exclusive to humans.

**Table 2:** Comparison of some basic characteristics of sexual approach behaviors in rodent and humans.

	Rats	Humans
Efficient stimulus	Olfactory + any other modality	Visual, auditory (fantasies)
Neutral stimuli may become sexual incentives through learning	Yes	Yes
Behavioral responses	Context dependent	Context dependent
Depends on gonadal hormones	Yes	Yes
Effects of SSRIs	May inhibit	May inhibit

Except for the two differences mentioned in the preceding paragraph, it seems that the mechanisms of sexual approach are most similar in these species. Unfortunately, the scarcity of drugs with established effect on human sexual approach make comparisons of drug effects extremely limited. Moreover, the complete absence of experimental evaluation of human sexual approach forces us to use genital responses to sexual stimuli as a proxy for actual approach as soon as we search for objective data. Nevertheless, the conclusion that sexual approach is controlled by similar behavioral and neural mechanisms in rats and humans seem warranted.

### Relationship Between Sexual Approach Behavior and Copulatory Behavior

In “Sexual Approach in Rodents” section, we have discussed sexual motivation, expressed as the intensity of genital responses in humans and approach to a potential mate in rodents. We also have mentioned that sexual motivation is a determinant of the intensity of copulatory behavior. A fundamental issue is whether sexual motivation is a unitary concept or not. If it is, then the intensity of genital responses and approach should always covary with the intensity of copulatory behavior. In humans, there does not seem to exist any systematic study of the relationship between genital responses to sexually relevant stimuli and the intensity of copulatory behavior. The typical setup for evaluating genital responses in men and women is such that no copulatory activity can occur in the testing situation. The only way to determine any possible relationship between the magnitude of the individual’s genital response and the intensity of copulatory behavior displayed by the same individual would be to enquire about the person’s sexual activity outside the laboratory. Under the conditions that magnitude of the genital response is stable between the laboratory and bedroom contexts and that the individual correctly reports his or her sexual activity, this might be an acceptable approximation. Unfortunately, this kind of study has not been performed, at least not published. Instead, much effort has been invested in finding out how genital responses relate to subjective sexual arousal, as discussed earlier. The issue of whether subjective arousal has any relationship to actual copulatory activities or if it is a useless concept has not been of much concern.

In rodents, there is direct experimental evidence showing that the intensity of copulatory behavior can be experimentally manipulated independently of the intensity of sexual approach behaviors (Ågmo, 2002). After repeatedly pairing ejaculation with a female smelling of fish oil with an injection of LiCl, a compound producing stomach ache and diarrhea, both approach to and copulation with such females were suppressed. However, the experimental males approached non-scented females with undiminished intensity, but they did not copulate with them when given the opportunity. Thus, the conditioned inhibition of approach was specific to an olfactory stimulus (fish oil) whereas inhibition of copulation generalized to any female. Pharmacological studies have also revealed that approach and copulation can be modified in opposite directions by drugs. The adrenergic α_2_ antagonist RX 821002 enhances approach behavior whereas copulation is reduced, for example (Chu and Ågmo, 2016a).

We have further evaluated the notion that approach can vary independently of copulation by calculating the correlation between sexual approach and copulatory behavior in a large number of rats. As shown in Table 3, there was no significant correlation between the intensity of approach and the intensity of copulatory behavior. Thus, at the level of the individual, there is no relationship between approach and copulation. However, if we instead look at the group level, for example comparing the mean intensity of sexual approach in a group of intact rats with that in a group of castrated males, we find a highly significant difference. The preference score was 0.70 ± 0.04 [mean ± standard error of the mean (SEM)] in the intact group vs. 0.46 ± 0.03 in the castrated group (*p* < 0.001). This is also the case with every aspect of copulatory behavior. In fact, the castrated males did not display a single mount, and obviously no intromission or ejaculation. Thus, the intact group shows a higher level of approach than the castrated group, and also a far more intense copulatory behavior.

**Table 3:** Pearson correlations between sexual approach behavior (quantified as a preference score^a^ obtained in the procedure described in Ågmo, 2003) and copulatory behavior in intact male rats having displayed at least one mount or one intromission in a test for copulatory behavior performed immediately after the test for approach behavior.

Copulatory behavior parameter	Correlation	*N*
Mount latency	0.016	195
Number of mounts	−0.009	195
Intromission latency	−0.067	173
Number of intromissions	0.064	173
Ejaculation latency	−0.008	154
Post-ejaculatory interval	−0.153	154

*^a^Preference score = [time spent approaching a sexually receptive female/(that time + the time spent approaching another male)]. Not all animals displaying mounts achieved intromission, and not all animals displaying intromission ejaculated during the test. The test duration was determined by the following criteria: end of the first post-ejaculatory interval, no intromission within 15 min of introduction of the female, no ejaculation within 30 min of the first intromission. Data were pooled from several experiments performed in the laboratory*.

Turning to females, we again find that there is no correlation between the intensity of approach and copulatory behavior in the divided cage, a procedure in which the female can pace sexual interaction (Table 4). As was the case with males, however, we find a clear relation between approach and copulation at the group level. When comparing data from ovariectomized females given either oil or EB + P, we find clear-cut differences both in approach and copulatory behavior. The mean ± SEM preference score was 0.72 ± 0.03 in hormone-treated females whereas it was 0.55 ± 0.06 in oil-treated females (*t*_(16)_ = 2.980, *p* = 0.009). The former displayed intense copulatory behavior whereas the latter showed none. It is evident that sexual approach at the individual level is unrelated to copulatory behavior, exactly as it is in males, but that there is a relationship at group level.

**Table 4:** Pearson correlations between sexual approach behavior and copulatory behavior in ovariectomized female rats given EB, 25 μg and P, 1 mg, 48 and 4 h before being subjected to a test for sexual approach behavior immediately followed by a test for copulatory behavior in the divided cage.

Copulatory behavior parameter	Correlation	*N*
Latency to enter male’s half	−0.156	30
Exit after male mount	0.010	29
Return latency after mount	−0.063	29
Exit after intromission	−0.300	25
Return latency after intromission	0.201	25
Return latency after ejaculation	0.056	24
Number of paracopulatory behaviors	−0.122	30

*Data are pooled from three separate experiments performed in the laboratory by Chiara Pozzato and AÅ. Approach behavior was quantified as a preference score established in the sexual incentive motivation test (Ågmo, 2003). Latency to enter male’s half, time between introduction of the female into her half and entry into the male half with all four paws; exit after mount, proportion of mounts followed by escape to the female’s half within 10 s; return latency after mount, time between the female’s entry into her half and her return, with all four paws, to the male’s half after having received a mount. Exit and return latency after intromission are self-explanatory. All females escaped after ejaculation. Thus, the proportion of exits is a constant and cannot be used for calculating a correlation*.

The contradictory observations between the lack of relationship between approach and copulation within the individual and the clear relationship at the group level is similar to the lack of relationship between serum testosterone concentration and the intensity of copulatory behavior at the individual level (e.g., Damassa et al., 1977, in rats and Brown et al., 1978, in men) even though testosterone is necessary for that behavior. This fact is normally explained by posing that above a minimum serum concentration of testosterone, further increases in concentration has no consequence. We propose that a similar principle also holds for sexual approach behavior. Although approach is a requisite for copulation, once the intensity of approach surpasses a certain level, further increase has no consequence.

## The Caveats

Female rat copulatory behavior is relatively straightforward, and relevant behavior patterns are limited to lordosis and paracopulatory behaviors. Other behaviors displayed by females, such as sniffing or anogenital sniffing of the male, have no relationship to copulatory behavior (Chu and Ågmo, 2014; Le Moëne and Ågmo, 2018). It can obviously be argued that rat lordosis has no equivalent in women, and that treatment effects on that behavior cannot be generalized to women. Nevertheless, the ease by which lordosis is displayed is determined by motivation, and since lordosis is a sexual response, that motivation is sexual. This same argument could be used for the paracopulatory behaviors. Most women are not ear-wiggling during copulation, yet rat ear-wiggling is, like any other behavior, controlled by motivation. Since this behavior is a response to a sexually relevant stimulus [a sexually active male induces far more ear-wiggling than a castrated male (Vreeburg and Ooms, 1985)], it can be supposed to be controlled by sexual motivation. Thus, any treatment effects on ear-wiggling represent effects on motivation. Even though the behavioral manifestations of sexual motivation are drastically different in rats and women there may well be similar underlying mechanisms in operation.

Likewise, in the male rat there is a series of behavioral parameters that have no equivalence in men. Rat measures such as the interval between intromissions or the proportion of mounts ending in vaginal penetration are probably meaningless. It is also uncertain whether these parameters represent motivation of any kind. The ease with which intromission is achieved depends on vascular erection as well as the activity in the penile striated muscles (Sachs, 1982; Giuliano et al., 1994). Any alteration in the coordinated activity of these processes can modify copulatory behavior, even though they are unrelated to sexual motivation. A complete description of male copulatory behavior might make it possible to distinguish effects on peripheral mechanisms from effects on motivation. However, any speculation about motivation based on copulatory behavior suffers from a considerable degree of uncertainty. It appears far more difficult and risky to infer changes in sexual motivation from changes in copulatory behavior in males than in females.

Sexual approach behaviors have sometimes been considered as the only acceptable indicator of sexual motivation. The data showing that the intensity of approach is unrelated to the intensity of copulatory behavior at the individual level makes this assertion somewhat exaggerated. If the proposal made above, that variations in the intensity of approach behaviors have no consequence for the intensity of copulatory behavior when the former are above some minimum level, is true it appears of limited interest to pursue treatments that might lead to enhancement beyond the minimum. To the contrary, in the case of search for treatments of hypoactive sexual desire disorder, it can be assumed that sexual approach behaviors in those affected are below the minimum, and any increase would consequently be beneficial. Thus, rodent models of human conditions involving reduced desire should evaluate approach rather than copulation.

The uncertainties regarding the correspondence between copulatory and sexual approach behaviors in rodents and humans are considerable, as mentioned. Nevertheless, there is no doubt that in both species, these behaviors are determined by one or another aspect of sexual motivation. We will now turn to an additional complication, jeopardizing any generalization from rodent to human. Whereas rodent sexual approach and copulation are mainly determined by preprogrammed mechanisms in the central nervous system, human sexual relationships are basically determined by social conventions. Anthropologists and sociologists have elegantly shown that human sexual behaviors, in the widest sense, are social constructions (Ford and Beach, 1951; Marshall and Suggs, 1971; Gagnon and Simon, 2002). Conventions determine to whom, when and where we can manifest sexual approach, how this approach should be manifested, and how to proceed in order to initiate copulatory activity. The nature of that activity, i.e., the motor patterns employed, are also largely determined by conventions, acquired through social learning. Even the impact of sexually relevant stimuli are made context-dependent because of social learning. A naked human body will not function as a sexual incentive on a nudist beach, whereas it usually is most efficient in the intimacy of a bedroom.

The fundamental role of social determinants in human sexual activities has obviously no equivalence in rodents. Insofar as those determinants influence the activation and behavioral manifestations of sexual motivation, rodent models are of limited help. However, even if social factors are crucial for human sexuality, there are basic neurobiological and behavioral mechanisms on which these factors act. According to the incentive motivation model presented in Figure 1, the central motive state prepares the ground for the actions of sexual incentives and for the responses to those. The incentives and the responses may well be heavily dependent on social learning, but without an appropriate central motive state no stimulus would act as an incentive and no response would be performed. There is no reason to believe that the nervous basis for the central motive state is drastically different in rodents and humans. This means that we should pursue means to discover the workings and manifestations of the activity of the central motive state underlying sexual motivation.

## Conclusion

Like any other behavior, sexual approach and copulation are determined by motivation. We assume that a particular motivational state makes organisms sensitive to sexually relevant stimuli which in turn activate responses, normally approach and copulation, eventually leading to sexual reward. This motivational state is called sexual motivation. It appears that there are some differences between the motivational state leading to the establishment of physical contact with a potential mate, approach, and the motivational state leading to the execution of copulatory acts. Since “motivational state” is an abstract concept, it must be anchored in reality through its behavioral manifestations. These manifestations can be rather different in rodents and humans, but the neurological underpinnings of the motivational state behind behavior are probably very similar. It should always be borne in mind that human sexual activities are a result of social learning and that we must go around the confound caused by this fact if we are to understand their motivational basis. This is often forgotten.

## Author Contributions

OLM and AÅ contributed equally to this manuscript.

## Conflict of Interest Statement

The authors declare that the research was conducted in the absence of any commercial or financial relationships that could be construed as a potential conflict of interest.
